# Supplementary material for: Novel Driver Strength Index highlights important cancer genes in TCGA PanCanAtlas patients
Source: PeerJ. 2022 Aug 11;10:e13860. doi: 10.7717/peerj.13860 (PMC9375969; doi:10.7717/peerj.13860)
Supplement: Supplemental Information 2 [file peerj-10-13860-s002.zip › COHORTS/genes plots/age/2021_8_16_14_9_distribution_cohorts_females_60-64.pdf]

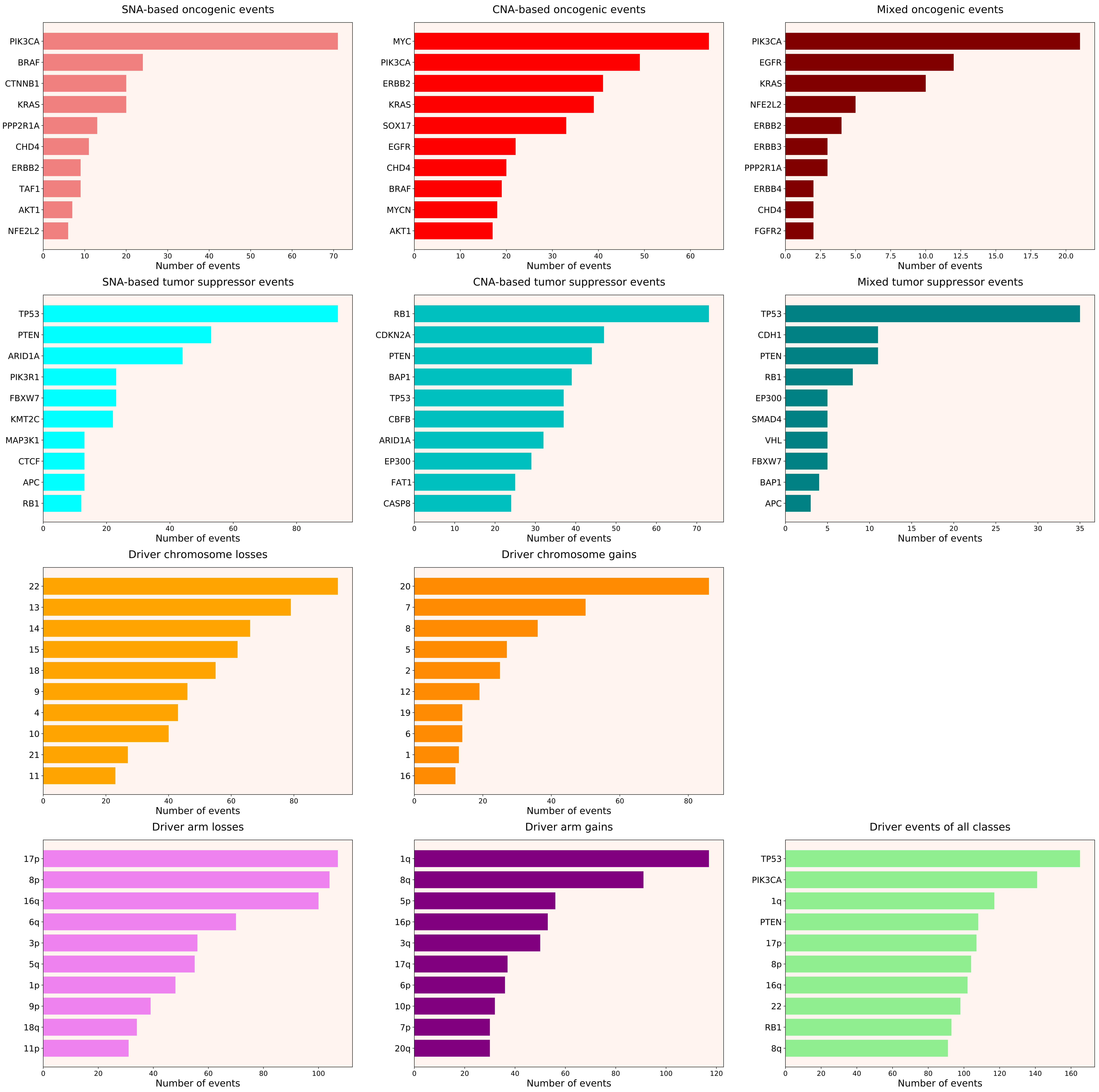
SNA-based oncogenic events
PIK3CA
BRAF
CTNNB1
KRAS
PPP2R1A
CHD4
ERBB2
TAF1
AKT1
NFE2L2
0 10 20 30 40 50 60 70
Number of events
CNA-based oncogenic events
MYC
PIK3CA
ERBB2
KRAS
SOX17
EGFR
CHD4
BRAF
MYCN
AKT1
0 10 20 30 40 50 60
Number of events
Mixed oncogenic events
PIK3CA
EGFR
KRAS
NFE2L2
ERBB2
ERBB3
PPP2R1A
ERBB4
CHD4
FGFR2
0.0 2.5 5.0 7.5 10.0 12.5 15.0 17.5 20.0
Number of events
SNA-based tumor suppressor events
TP53
PTEN
ARID1A
PIK3R1
FBXW7
KMT2C
MAP3K1
CTCF
APC
RB1
0 20 40 60 80
Number of events
CNA-based tumor suppressor events
RB1
CDKN2A
PTEN
BAP1
TP53
CBFB
ARID1A
EP300
FAT1
CASP8
0 10 20 30 40 50 60 70
Number of events
Mixed tumor suppressor events
TP53
CDH1
PTEN
RB1
EP300
SMAD4
VHL
FBXW7
BAP1
APC
0 5 10 15 20 25 30 35
Number of events
Driver chromosome losses
22
13
14
15
18
9
4
10
21
11
0 20 40 60 80
Number of events
Driver chromosome gains
20
7
8
5
2
12
19
6
1
16
0 20 40 60 80
Number of events
Driver arm losses
17p
8p
16q
6q
3p
5q
1p
9p
18q
11p
0 20 40 60 80 100
Number of events
Driver arm gains
1q
8q
5p
16p
3q
17q
6p
10p
7p
20q
0 20 40 60 80 100 120
Number of events
Driver events of all classes
TP53
PIK3CA
1q
PTEN
17p
8p
16q
22
RB1
8q
0 20 40 60 80 100 120 140 160
Number of events
